# Undifferentiated pleomorphic sarcoma with osteoclast-like giant cells of the female breast

**DOI:** 10.1186/1477-7819-11-21

**Published:** 2013-01-26

**Authors:** Giancarlo Balbi, Luca Di Martino, GianPaolo Pitruzzella, Diego Pitruzzella, Flavio Grauso, Antonella Napolitano, Elisabetta Seguino, Francesco Gioia, Pasquale Orabona

**Affiliations:** 1 U.O. Ginecologia ed Ostetricia, Seconda Università degli Studi di Napoli, c/o S. Anna e S. Sebastiano Hospital, Caserta, Italy; 2 U.O.S.D. Breast Unit, S. Anna e S. Sebastiano Hospital, Caserta, Italy; 3 U.O.C. Anatomic Pathology, S. Anna e S. Sebastiano Hospital, Caserta, Italy; 4 Department Obstetrics and Gynaecology and Reproduction, University of study of Naples, Largo Madonna Delle Grazie, 1, Napoli 80138, Italy

**Keywords:** Breast neoplasm, Female, Immunohistochemistry, Sarcoma

## Abstract

The authors describe a case of undifferentiated pleomorphic sarcoma of the breast occurring in a 50-year-old woman who presented with a palpable mass in her right breast. She first noticed the mass one month previously. Core needle biopsy showed connective tissue including epithelioid and spindle cells. The patient underwent total mastectomy without axillary lymph node dissection. Based on examination of the excised tumor, the initial pathologic diagnosis was atypical spindle-shaped and ovoid cells with uncertain malignant potential. Histological findings with immunomarkers led to the final diagnosis of undifferentiated pleomorphic sarcoma.

This case highlights a rare and interesting variant of primary breast sarcoma and the important role of immunohistochemistry in defining histological type and differential diagnosis. Hence, undifferentiated pleomorphic sarcoma has been a diagnosis of exclusion performed through sampling and critical use of ancillary diagnostic techniques.

## Background

Breast sarcomas are histologically heterogeneous nonepithelial malignancies that arise from the connective tissue within the breast [1]. They are relatively uncommon and represent less than 1< of all primary breast malignancies.

Almost every previous reference to this entity in the medical literature is in the form of isolated case reports. In all cases, the patients had been diagnosed clinically as having breast carcinoma and the correct tissue diagnosis was established histologically. The prevalence, risk factors and clinical course of breast sarcomas are less well characterized than breast tumors arising from epithelial tissue. Herein, we report a case of primary undifferentiated pleomorphic sarcoma in a 50-year-old woman; this case highlights a rare and interesting variant of primary breast sarcoma and the diagnostic difficulty that physicians and pathologists may encounter with it.

## Case presentation

A 50-year-old woman presented to the Breast Unit of Sant’ Anna and San Sebastiano Hospital of Caserta, Italy, after becoming aware of a non-tender lump in the median areolar area of her right breast. She first noticed the mass one month previously. During this period, she did not have any pain or discharge from the nipple. Further, she had no family history of malignancy, including breast cancer. She was in antihypertensive therapy.

She had never undergone any radiation and hormone replacement therapy. On physical examination, the patient had a demarcated, mobile, firm and fast-growing mass in her right breast.

The mass was not tender, approximately 4 cm in diameter, and was detected in the median areolar area of the right breast. There was no clinical evidence of regional lymphadenopathy.

**Figure 1:**
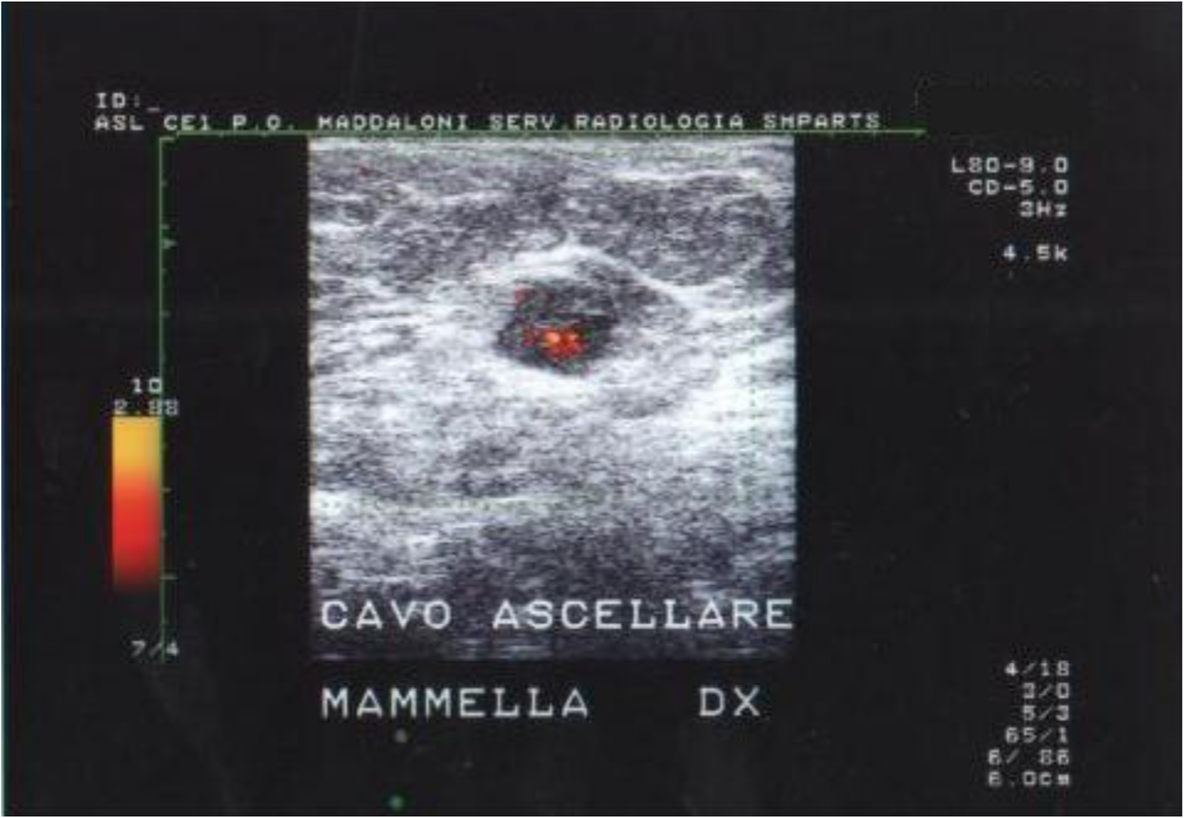
Opaque and ovoid lesion occupying mediant equatorial region of the right.

Mammography revealed an opaque and ovoid lesion occupying the median equatorial region of the right breast; this lesion was 35 mm in diameter, with regular margins, moderate intensity and homogeneous density; there was no evidence of defined focus of pathological calcification (Figure 1). Ultrasonography revealed a bulky, firm, hypoechoic lesion, with blurred and irregular margins, richly vascularized on color Doppler, with high speed systolic flow. This mass was 45 mm in diameter, and referred to as a heteroplastic lesion. There was no evidence of lymphadenopathy.

Ultrasound-guided fine needle aspiration on the lesion showed epithelioid and spindle cells, which were positive only to the immunohistochemical staining for vimentin and so we suspected a mesenchymal lesion.

An ultrasound-guided core needle biopsy performed subsequently showed connective tissue, including cells similar to those already present in the needle aspirate and there were many multinuclear osteoclast-like giant cells. The immunohistochemical staining confirmed the mesenchymal nature of the lesion (positivity for vimentin), but did not indicate clearly a specific histotype for the actin, CD34 and S100 protein negativity.

Preoperative examination consisted of a complete blood count, serum kidney and liver function, thyroid function test, and tests for the levels of several hormones related to the development of gynecomastia, including estrogen, testosterone, prolactin and gonadotrophic hormones as well as cancer markers (CEA, CA 15–3, CA 125). Ultrasound (US) of the abdomen failed to document any abnormalities. An isotope bone scan did not reveal any abnormal uptake. All results were within the normal limits.

The patient underwent total mastectomy without axillary lymph node dissection, awaiting histology.

Macroscopic examination of the specimen, measuring 19 × 15 × 8 cm and including the normal nipple, revealed a grayish, apparently delimited nodular lesion, measuring 10 cm in diameter, with cystic-necrotic and few hemorrhagic areas. The lesion was 0.5 cm away from the fascia and 1 cm away from the skin; furthermore, another grayish nodular lesion measuring 1.5 cm was in the subcutaneous tissue.

**Figure 2:**
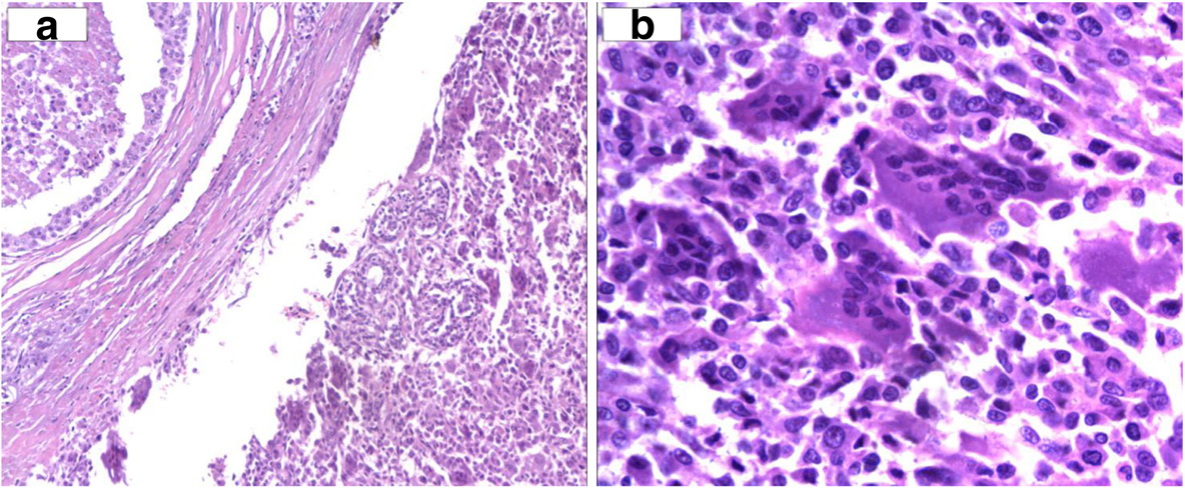
**a: Breast tissue: invasive neoplasm adjacent to dilacted duct. **(Hematoxylin Eosin 20 x). **b**: Detail of the neoplastic population(Hematoxylin Eosin 40 x).

**Figure 3:**
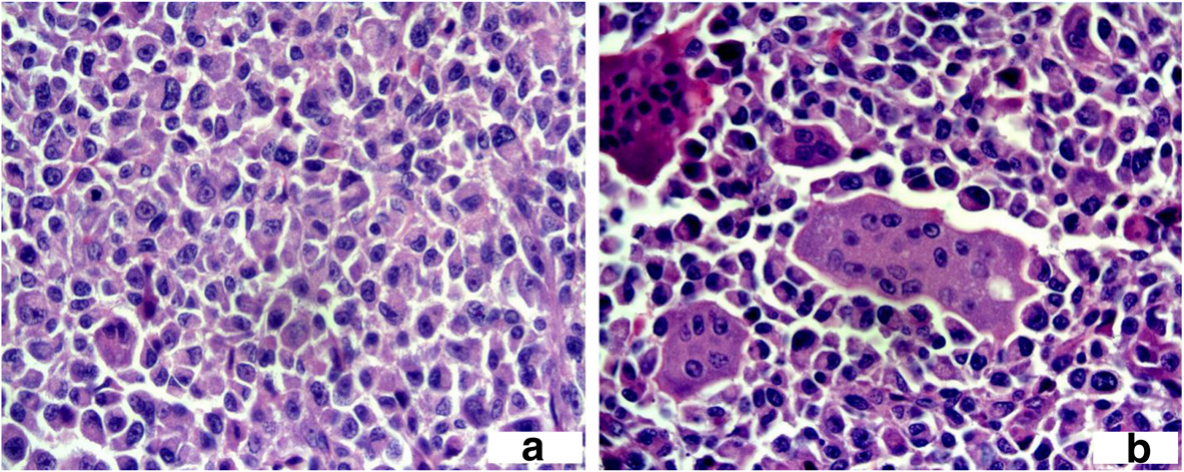
**a: Three multinuclear giant cells surrounded by neoplastic mononuclear cells (Hematoxylin Eosin 40 x). b**: neoplastic cells nuclear features (Hematoxylin Eosin 40 x).

Microscopic examination of the sections from the specimen showed nodular proliferation of pleomorphic-spindle-shaped and ovoid cells, with connective tissue septa, mixed with many multinuclear osteoclast-like giant cells that did not show, mainly, malignant features (Figures 2a-2b; Figure 3a, 3b).

**Figure 4:**
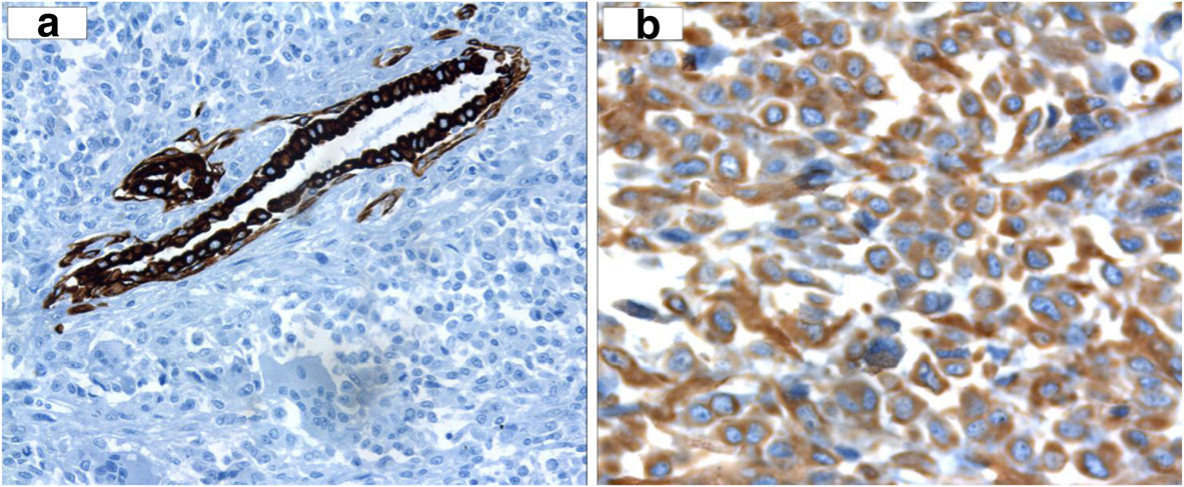
**a: Immunohistochemical staining for Cytokeratin Pan stains the ductal structure while tumor cells are negative(20 x). b**: Positivity for Vimentin(40 x).

Necrotic areas and abnormal mitosis were identified. Immunohistochemical stainings for Cytokeratin Pan (Figure 4a), Cytokeratin 7-8-18-19, EMA (epithelial membrane antigen), S100 protein, SMA (smooth muscle actin) and CD34 were negative; immunohistochemical staining for vimentin was positive (Figure 4b). Mitotic activity was observed with a high proliferation index, assessed with Ki67. Moreover, in the surrounding parenchymal component, there were multiple foci of intraductal carcinoma, evaluated with immunohistochemical staining for muscle actin and collagen IV. The lesion was contained within the margins of excision.

The histological and immunohistochemical findings established the diagnosis of undifferentiated pleomorphic sarcoma with osteoclast-like giant cells of the breast.

The patient was monitored after the operation and we did not consider that adjuvant treatment was necessary in the presence of adequate local control and in the absence of metastatic spread of the disease. Follow-up mammography has been satisfactory to date, and she remained well without tumor recurrence at 15 months.

## Discussion and conclusion

Primary breast sarcoma is a rare type of cancer arising from the mesenchymal tissue of the breast; it accounts for less than one percent of all breast malignancies [2].

Undifferentiated pleomorphic sarcoma has been rarely seen in the breast [3] and, in the previous reports, it consisted of 10.5 to 24< of all primary breast sarcomas [3,4].

Most undifferentiated pleomorphic sarcomas of the breast affect mainly advanced-aged women (over 64 years of age), but it has also been described in men [5]. However, it is very difficult to diagnose mammary undifferentiated pleomorphic sarcoma from the clinical features, such as those revealed by palpation, mammogram and US appearance. Concerning ultrasound examination and mammography, there are not specific features to obtain a differential diagnosis between sarcomas. Neither the symptoms nor the physical findings suggest the diagnosis.

Immunohistochemistry is essential, primarily, to distinguish undifferentiated carcinoma from mesenchymal neoplasia and, subsequently, after the exclusion of epithelial neoplasia, it is necessary to define the histogenesis of the lesion [6].

Immunohistochemical studies in our case were performed on the tissue block containing proliferating tumoral cells. The tumoral cells stained intensely for vimentin (a mesenchimal marker), but no immunoreactivity for the Cytokeratin Pan (epithelial cells marker), Leukocyte Common Antigen (lymphoid cells marker), SMA (smooth muscle actin), Desmin (smooth and streaked muscular cells marker), S100 (neuronal cells marker), CD34 and bcl-2 (to identify malignant phylloides tumor), CD99 (synovial sarcoma marker) was detected.

Imaging methods and macroscopy have revealed well-circumscribed masses with heterogeneous composition. Further, they have been identified as pale fibrous and fleshy areas admixed with zones of (cystic) necrosis, hemorrhage or myxoid features [7].

Microscopically, lesions exihibit cells showing marked pleomorphism admixed with bizarre giant cells, spindle cells and variable foamy cells [8]. A storiform growth pattern and variable chronic inflammatory cells are also common [7].

Limited data on undifferentiated pleomophic sarcoma indicate an aggressive clinical course and high incidence of recurrence and metastasis. Overall, five-year survival of patients with undifferentiated pleomophic sarcoma has been roughly 50<[7]. Axillary dissection has been generally considered unnecessary for undifferentiated pleomophic sarcoma of the breast, since these tumors rarely spread through the lymphatic system [2,9]. Therefore therapeutic treatment of these tumors is mastectomy without lymphoadenectomy. The literature’s data show approximately 40< local recurrence and that 60< of the cases develop distant metastases [10]. The role of adjuvant chemotherapy and radiation also has been unclear [2,9].

In our case, the patient underwent a simple total mastectomy of the right breast, not including axillary lymph node dissection.

In the presence of a partial epithelial differentiation, the basic requirement for the diagnosis of a primary sarcoma of the breast is the exclusion of epithelial origin (axillary lymph node dissection is necessary) but the wide sampling we performed and the immunohistochemical negativity for cytokeratins and EMA exclude this diagnosis.

In our patient, we found multiple foci of intraductal carcinoma but sarcomatous components were the prevailing neoplastic element of the lesion, thus subcategorizing the tumor under the sarcoma type with typical biological behavior and therapeutic approach [11].

Differential diagnosis should include metaplastic carcinoma (sarcomatoid), malignant phyllodes tumor, inflammatory myofibroblastic tumor (IMT), myxofibrosarcoma, stromal sarcoma, leiomiosarcoma, rhabdomyosarcoma and liposarcoma.

Metaplastic carcinoma is a general term used to indicate breast tumor, in which the predominant component has a different feature than epithelial or typical ductal. It is a rare heterogeneous neoplasm generally characterized by a mixture of adenocarcinoma with dominant areas of spindle cells, squamous and other mesenchymal differentiation [12]. There are several variants: 1) Ssarcomatoid carcinoma characterized by malignant struma of fibrohistiocitoma type (pleomorphic sarcoma-like), chondrosarcoma, osteosarcoma, rhabdomyosarcoma, angiosarcoma or a combination of these histotypes. In the stromal component, the immunohistochemical strainings show positivity for vimentin and, variably, for pan-cytocheratin and cytocheratin 7. 2) Spindle cell carcinoma: epithelial component is invasive or intraductal. Spindle cells may not be represented and so simulate a fibromatosis or a low grade fibroblastic sarcoma with the presence of copious stromal collagen. 3) Carcinoma with osteoclastic giant cells enters into the differential diagnosis with our case, but also in this variant stromal component shows positivity for epithelial markers. 4) Squamous cell carcinoma: the main problem is the differentiation with a metastatic squamous cell carcinoma, which requires a careful correlation with clinical data and medical history, in addition to the immunochemistry. In some cases of metaplastic carcinoma, immunohistochemical staining for cytocheratins may be negative and so we can add staining with EMA. These forms of neoplasm usually have a more aggressive behavior than ductal carcinoma.

Malignant phyllode tumors consist of a predominant mesenchymal component and a benign epithelial component [6]. According to stromal features, there are three subgroups: benign, borderline and malignant lesions. Malignant lesions, macroscopically, show infiltrative growth and necrotic and hemorrhagic areas. There is histologically the predominance of a mesenchimal component rather than an epithelial, nuclear pleomorphism, high mitotic index and necrosis. The stromal features could simulate pleomorphic sarcoma, liposarcoma, chondrosarcoma or, exceptionally, rhabdomyosarcoma. The diagnosis of malignant forms, in which giant cells may be present, requires an extended sampling to demonstrating the presence of a ductal involvement. Immunochemistry usually points out the positivity of stromal cells for CD34 and bcl-2 in 25< of the cases.

IMT is a rare spindle cell neoplasm occurring in the young, characterized by the presence of many inflammatory elements mixed with mesenchymal cells. Histological features consist of spindle or polygonal cells, admixed with fibroblasts and myofibroblasts, plasmacells and lymphocytes in various proportions.

An IMT is a low-grade neoplastic lesion showing lack of atipia, hyperchromasia and abnormal mitotic figures [13]. Immunohistochemistry showed positivity of the spindle cells for vimentin and, variably, for muscle actin and smooth muscle actin. The positivity for Cytokeratin AE1/AE3 and ALK is variable. The tumor can be differentiated from undifferentiated pleomorphic sarcoma by the absence of necrosis and of marked nuclear pleomorphism, low mitotic index and a different immunohistochemical profile.

Myxofibrosarcoma, previously designated as malignant myxoid fibrous histiocytoma, is the most common sarcoma in adults and it commonly occurs in people 60 years old or older. Histologically, in a low grade lesion the neoplasm shows a uniform myxoid matrix and few nuclear atypia. In high grade lesions, the tumor shows pleomorphism with frequent mitotic figures and large necrosis areas. Immunohistochemical staining shows positivity for vimentin and variably for CD34, and negativity for cytokeratin, S-100 and actin. The neoplasm can be distinguished from undifferentiated pleomorphic sarcoma by the uniform presence of myxoid degeneration areas [14].

Another malignant lesion to be considered in the differential diagnosis is the stromal sarcoma that originates from the stroma of the breast. It is completely devoid of epithelial components, unlike the phyllodes tumor. Macroscopically the lesion appears solid, grayish-white and homogeneous with the presence of necrosis’ foci. Histologically it is characterized by a proliferation of monomorphic spindle cells but sometimes cytologic atypia and high mitotic activity with infiltrative growth have been reported. Immunohistochemical staining shows strong vimentin reactivity. Of note, areas with stromal origin in the breast may be focally immunoreactivate for CD10.

Immunohistochemistry plays a major role in differentiating pleomorphic sarcomas from other breast mesenchymal malignancies: leimiosarcomas, liposarcomas and rhabdomyosarcomas. The leiomyosarcomas are very rare: 30 cases have been reported in the literature and the forms of higher degree may have marked nuclear pleomorphism, high mitotic index and necrosis. This lesion shows diffuse positivity for actin and muscle desmin which are absent or minimal in pleomorphic sarcoma. The primary rhabdomyosarcomas are more frequently found in association with malignant epithelial areas of a metaplastic carcinoma or undifferentiated lesions of malignant phyllodes tumor. Cases of pure rhabdomyosarcoma of the breast are extremely rare (about 20 in the Armed Forces Institute of Pathology case series), are typical of young women (under 40 years old) and show strong actin and desmin reactivity but also diffuse positivity to the immunohistochemical staining for myoglobin and myogenin. The liposarcomas and pleomorphic liposarcomas are extremely rare. The second are characterized by proliferation of hypoblasts and demonstration of immunohistochemical positivity for MUM1 [15].

Breast sarcoma should also be distinguished from benign spindle cell lesions, such as myofibroblastoma, nodular fasciitis and fibromatosis [16-18]. The fibromatosis are rare mesenchymal breast lesions, sometimes caused by previous breast implants and local recurrence. They are characterized by infiltrative growth and by monomorphic spindle cell proliferation, with a low mitotic index and occasional presence of eosinophil bodies. Compared with pleomorphic sarcoma, the fibromatosis show the monomorphic cellular component and the positivity of immunohistochemical staining for beta catenin. The nodular fasciitis is clinically characterized by painful injury with a rapid onset and growth. It appears as a circumscribed nodular lesion and histologically it has been identified by areas of collagen tissue, foci of mucoid degeneration, hemorrhagic extravasation, lymphocytes and multinucleated giant cells. The typical cells are myofibroblasts, with mild nuclear pleomorphism and numerous mitoses. Immunohistochemical evaluation shows positivity for vimentin, actin and CD10.

In our case, the tumor did not arise from the epithelial tissue but from the mesenchymal tissue of the breast, being consistent with the negative immunoreactivity for CK, EMA, SMA, desmin and S-100 proteins.

Histologic findings of many atypical cells and abnormal mitotic activity, a negative immunoreactivity for SMA and ALK helped to exclude IMT from the differential diagnostic consideration. This case was distinguished from myofibroblastoma by negative immunoreactivity for SMA, CK, EMA, CD34, desmin and S100 protein.

Instead, osteosarcoma can be classified into three subtypes, including fibroblastic, osteoblastic and osteoclastic osteogenic sarcomas. Histological differentiation is important, since fibroblastic osteogenic sarcomas are associated with a better survival outcome than other pathological types [19,20]. The presence of bone or osteoid elements in breast lesions is relatively rare, but has also been described in epithelial neoplasms, such as metaplastic carcinomas. Metaplastic carcinomas typically are immunoreactive for cytokeratin, identifying them as epithelial neoplasms [19]. The basic requirements for the diagnosis of a primary osteosarcoma of the breast, according to Allan and Soule, include (a) the presence of neoplastic osteoid or bone, (b) the exclusion of origin in the bone, and (c) the absence of an epithelial component [21]. In our patient, the result of an isotope skeletal bone scan aided in excluding the possibility of a primary bone tumor, while histological and immunohistochemical analyses showed no evidence of epithelial differentiation.

Although histological features of the removed mass were suggestive of a malignant pleomorphic spindle cell tumor, the immunohistochemical responses failed to disclose the line of tumoral differentiation. Hence, the final diagnosis of the tumor was undifferentiated pleomorphic sarcoma.

In conclusion, undifferentiated pleomorphic sarcoma of the breast is an uncommon mesenchimal neoplasm that presents diagnostic challenges. Mammography and breast ultrasonography showed a breast lump, but features were often not diagnostic.

It has been a diagnosis of exclusion performed through sampling and critical use of ancillary diagnostic techniques.

Therefore, as with all rare tumors, undifferentiated pleomorphic sarcoma should be managed in reference centers to determine whether treatment of choice is surgical excision or total mastectomy, and whether some form of adjuvant therapy can have beneficial effects.

## Consent

Written informed consent was obtained from the patient for publication of this report and any accompanying images.

## Abbreviations

EMA: Epithelial Membrane Antigen; SMA: Smooth Muscle Actin; IMT: Inflammatory Myofibroblastic Tumor; ALK: Anaplastic Lymphoma kinase; MUM: Multiple Myeloma Oncogene; CK: Cytokeratin.

## Competing interest

No authors have conflict of interest with any financial organization regarding the material discussed in the manuscript and all authors contribute equally.

## Authors’ contributions

All authors equally contribute to manuscript and they read and approve it.
